# Cross-Genera PCR Amplification of DNA from Apicomplexan Parasites

**Published:** 2018-09-30

**Authors:** Philippe Gil de Mendonça

**Affiliations:** Institute of Comparative Tropical Medicine and Parasitology, Ludwig Maximilian University, Munich, Germany

**Keywords:** Babesia, Theileria, Hepatozoon, Toxoplasma, Hammondia

## Abstract

**Background:**

The discovery of an unexpected genetic sequence raised doubts about the specificity of a primer pair targeting *Babesia* spp. and *Theileria* spp. This study aimed to check the specificity of this primer pair.

**Methods:**

Conventional end-point PCR and real-time PCR protocols using primers targeting the 18S rRNA gene V4 hypervariable region of *Babesia* spp. and *Theileria* spp. were tested for potential cross-genera amplification using DNA from a palette of parasitic protists and pathogenic bacteria as a template. These investigations took place at the Ludwig Maximilian University of Munich (Germany) in 2010 as part of the EDEN project.

**Results:**

Successful amplification was obtained with DNA from five apicomplexan genera: *Babesia*, *Theileria*, *Hepatozoon*, *Toxoplasma*, and *Hammondia*. No amplicons were obtained when DNA from *Leishmania infantum* or bacteria within the genera *Borrelia*, *Leptospira* or *Anaplasma* was used as a template.

**Conclusion:**

This cross-genera amplification ability is useful for the quick exclusion of many parasite species from PCR negative diagnostic samples. Accurate species identification from PCR positive samples requires genetic sequencing of the amplicon.

## Introduction

*Babesia* spp. and *Theileria* spp. are widespread tick-borne parasites. They are a cause of concern to the medical and veterinary community worldwide (1–10). Several species are even considered as an emerging threat to human health (9–10). The EU-funded EDEN project focused specifically on investigating emerging diseases (11). Within this context, arthropod vectors and vertebrate hosts were routinely sampled and screened for a large palette of zoonotic pathogens. A combination of techniques (12), namely PCR followed by reverse-line blot (RLB), was routinely used for screening *Babesia* spp. and *Theileria* spp. On various occasions, the panel of RLB probes available was not suitable for the identification of the parasite species involved. In such cases, amplicons were sequenced. Sequencing revealed the presence of *Sarcocystis*-like organisms (sequences did not match any known species in GenBank, and identity values were relatively low). These results raised serious concerns about the specificity of the PCR protocol used for screening *Babesia* spp. and *Theileria* spp.

In order to check for the actual presence of further cases of cross-genera amplification, the tests described below were performed using template DNA from a variety of protists and other microorganisms.

## Materials and Methods

PCR was performed with DNA from reference samples of parasitic protists as a template. Reference samples of parasitic protists included: *Babesia canis*, *B. capreoli*, *B. diver-gens*, *B. gibsoni*, *B. microti* (syn. *Theileria microti*), *B. rodhaini* (syn. *Theileria rodhaini*), *B. vogeli*, *Hepatozoon sylvatici*, *Toxoplasma gondii*, *Hammondia hammondi* and *Leishmania infantum*.

PCR was also performed with DNA from wildlife samples (arthropod vectors and vertebrate hosts) known to be positive for a palette of bacterial pathogens. These bacterial pathogens included *Borrelia afzelii*, *Leptospira interrogans* and *Anaplasma phagocytophilum*. Pure water was used as no-template control.

DNA was amplified using primers (F 5′-GACACAGGGAGGTAGTGACAAG and R 5′-CTAAGAATTTCACCTCTGACAGT) targeting the 18S rRNA gene V4 hypervariable region of *Babesia* spp. and *Theileria* spp. (13).

Real-time PCR with dissociation curve analysis was performed with QuantiFast SYBR^®^ Green (QIAGEN, Hilden, Germany) following the manufacturer’s instructions.

Conventional end-point PCR followed a modified touch-down protocol (14). One cycle at 95 °C for 5min for Hot Start Taq activation was followed by touch-down steps (denaturation at 94 °C for 20sec, annealing for 30 sec starting at 67 °C and dropping to 57 °C at a rate of 1 °C/step, extension at 72 °C for 30sec) prior to 40 amplification cycles (denaturation at 94 °C for 20sec, annealing at 57 °C for 30sec, extension at 72 °C for 30 sec). Reaction mix composition was: 5.00μl 10× QIAGEN PCR buffer (at 15mM MgCl_2_), 2.00μl QIAGEN MgCl_2_ (at 25mM), 1.00μl QIAGEN dNTPs (10mM), 1.00μl of each primer (10μM), 29.75μl ultra-pure water, 0.25μl QIAGEN Hot Start Taq Plus (5U/μl). Total volume was 50μl, including 10μl template DNA. PCR products were separated by electrophoresis through 1.5% agarose gels stained with GelRed® (Biotium, Hayward, California). Size markers (reference DNA ladder) were included in each gel. Amplicons and size markers were visualized by UV transillumination. Amplicon sizes were estimated with GelAnalyzer 2010 (www.gelanalyzer.com/download.html).

These investigations took place at Ludwig Maximilian University, Munich, Germanyin 2010 as part of the EDEN project.

Sequencing of amplicons was performed by Eurofins MWG GmbH (Ebersberg, Germany).

## Results

As expected, all *Babesia* spp. and *Theileria* spp. yielded amplicons with strong positive signals on both conventional and real-time PCR, and their sequences were consistent with data available on GenBank, thus confirming successful and accurate PCR amplification of *Babesia* and *Theileria* reference samples.

*Hepatozoon sylvatici*, *Toxoplasma gondii*, and *Hammondia hammondi* also yielded amplicons with strong positive signals on both conventional and real-time PCR, and their sequences were also consistent with data available on GenBank, thus also confirming successful and accurate PCR amplification of *Hepatozoon*, *Toxoplasma* and *Hammondia* reference samples.

No amplicon could be obtained from *L. infantum*. No amplicon was obtained either from any of the bacterial pathogens tested.

Melting temperature values for the various species successfully amplified ranged from 79.8 °C to 85.7 °C with overlap between various species. Amplicon sizes ranged from 402bp to 528bp with overlap between various species.

## Discussion

The taxonomy of protists has undergone many revisions and has been revolutionized from top to bottom in the last few years (15, 16). The taxonomy of the species within the genus *Babesia* is no exception to this rule (17, 18). However, for the sake of simplicity, a traditional approach was retained in the present article which is concerned with molecular diagnostic methodology, rather than taxonomic changes.

The PCR primer pair used in the present study was known for its ability to detect two closely related protist genera: *Babesia* and *Theileria*. The above-mentioned results revealed the ability to detect yet further protist genera, namely *Hepatozoon* and the closely related *Toxoplasma* and *Hammondia*.

All these genera belong to the Apicomplexa, which suggests that the PCR primer pair used here might amplify even more genera within this phylum. This unexpected widespread cross-genera amplification ability might seem problematic at first glance, however, this turns out to be a rather useful property when considered in a wider context. Indeed, screening with this primer pair makes it possible to exclude a large number of parasitic species at once for those samples yielding negative PCR results, thus avoiding expansive and time-consuming further testing for the numerous species within the genera known to be efficiently amplified by this peculiar primer pair. As a corollary to this, it will be most useful to perform yet further testing of additional genera within the Apicomplexa in order to establish the full width of the palette of genera efficiently amplified and thus detected when screening samples with this primer pair. Additional testing of protist genera outside the Apicomplexa would also be desirable.

Most importantly, due to widespread overlaps in amplicon sizes and melting temperatures on the one hand, and due to limitations of the RLB technique on the other hand, genetic sequencing of the amplicon is required for accurate species identification.

## Conclusion

The range of detection of the primer pair targeting the 18S rRNA gene V4 hypervariable region of *Babesia* spp. and *Theileria* spp. is not limited to these two closely related genera. At least three additional apicomplexan genera are efficiently amplified by this primer pair. These genera are *Hepatozoon*, *Toxoplasma*, and *Hammondia*. Genetic sequencing of the amplicons is required for accurate species identification.
